# A nutrient-wide association study for risk of prostate cancer in the European Prospective Investigation into Cancer and Nutrition and the Netherlands Cohort Study

**DOI:** 10.1007/s00394-019-02132-z

**Published:** 2019-11-08

**Authors:** Nikos Papadimitriou, David Muller, Piet A. van den Brandt, Milan Geybels, Chirag J. Patel, Marc J. Gunter, David S. Lopez, Timothy J. Key, Aurora Perez-Cornago, Pietro Ferrari, Paolo Vineis, Elisabete Weiderpass, Heiner Boeing, Antonio Agudo, María-José Sánchez, Kim Overvad, Tilman Kühn, Renee T. Fortner, Domenico Palli, Isabel Drake, Anders Bjartell, Carmen Santiuste, Bas H. Bueno-de-Mesquita, Vittorio Krogh, Anne Tjønneland, Dorthe Furstrand Lauritzen, Aurelio Barricarte Gurrea, José Ramón Quirós, Pär Stattin, Antonia Trichopoulou, Georgia Martimianaki, Anna Karakatsani, Elin Thysell, Ingegerd Johansson, Fulvio Ricceri, Rosario Tumino, Nerea Larrañaga, Kay Tee Khaw, Elio Riboli, Ioanna Tzoulaki, Konstantinos K. Tsilidis

**Affiliations:** 1 Department of Hygiene and Epidemiology, University of Ioannina School of Medicine, Ioannina, Greece; 2 Department of Epidemiology and Biostatistics, School of Public Health, Imperial College London, St Mary’s Campus, London, W2 1PG UK; 3 Department of Epidemiology, GROW School for Oncology and Developmental Biology, Care and Public Health Research Institute (CAPHRI), Maastricht University, Maastricht, The Netherlands; 4 Department of Biomedical Informatics, Harvard Medical School, Boston, MA USA; 5 International Agency for Research on Cancer, Lyon, France; 6 Department of Preventive Medicine and Community Health, UTMB School of Medicine, Galveston, TX USA; 7 Division of Urology, UTHealth McGovern Medical School, Houston, TX USA; 8 Nuffield Department of Population Health, Cancer Epidemiology Unit, University of Oxford, Oxford, UK; 9 Italian Institute for Genomic Medicine (IIGM), Turin, Italy; 10 Department of Epidemiology, German Institute for Human Nutrition Potsdam-Rehbrücke, Nuthetal, Germany; 11 Unit of Nutrition and Cancer, Cancer Epidemiology Research Program, Catalan Institute of Oncology-IDIBELL, L’Hospitalet de Llobregat, Barcelona, Spain; 12 Escuela Andaluza de Salud Pública, Granada, Spain; 13 Universidad de Granada. ibs.GRANADA, Granada, Spain; 14 CIBER de Epidemiología y Salud Pública (CIBERESP), Madrid, Spain; 15 Department of Public Health, Aarhus University, Aarhus, Denmark; 16 Division of Cancer Epidemiology, German Cancer Research Center (DKFZ), Heidelberg, Germany; 17 Cancer Risk Factors and Life-Style Epidemiology Unit, Institute for Cancer Research, Prevention and Clinical Network, ISPRO, Florence, Italy; 18 Department of Clinical Sciences in Malmö, Lund University, Malmö, Sweden; 19 Department of Urology, Lund University, Malmö, Sweden; 20 Department of Clinical Sciences, Lund University, Malmö, Sweden; 21 Department of Epidemiology, Murcia Regional Health Council, IMIB-Arrixaca, Murcia, Spain; 22 Department for Determinants of Chronic Diseases (DCD), National Institute for Public Health and the Environment (RIVM), Bilthoven, The Netherlands; 23 Department of Gastroenterology and Hepatology, University Medical Centre, Utrecht, The Netherlands; 24 Department of Social and Preventive Medicine, Faculty of Medicine, University of Malaya, Kuala Lumpur, Malaysia; 25 Epidemiology and Prevention Unit, Fondazione IRCCS Istituto Nazionale dei Tumori di Milano, Milan, Italy; 26 Diet, Genes and Environment, Danish Cancer Society Research Center, Copenhagen, Denmark; 27 Department of Public Health, Faculty of Health and Medical Sciences, University of Copenhagen, Copenhagen, Denmark; 28 Navarra Public Health Institute, Pamplona, Spain; 29 Navarra Institute for Health Research (IdiSNA), Pamplona, Spain; 30 Public Health Directorate, Asturias, Spain; 31 Department of Surgical Sciences, Uppsala University, Uppsala, Sweden; 32 Hellenic Health Foundation, Athens, Greece; 33 WHO Collaborating Center for Nutrition and Health, Unit of Nutritional Epidemiology and Nutrition in Public Health, Department of Hygiene, Epidemiology and Medical Statistics, School of Medicine, National and Kapodistrian University of Athens, Athens, Greece; 34 2nd Pulmonary Medicine Department, School of Medicine, National and Kapodistrian University of Athens, “ATTIKON” University Hospital, Haidari, Greece; 35 Department of Medical Biosciences, Pathology, Department of Biobank Research, Umeå University, Umeå, Sweden; 36 Departments of Odontology, Section of Cardiology, Biobank Research, Public Health and Clinical Medicine, Umeå University, Umeå, Sweden; 37 Department of Clinical and Biological Sciences, University of Turin, Orbassano, Italy; 38 Cancer Registry and Histopathology Unit, “M.P.Arezzo” Hospital, Ragusa, Italy; 39 Epidemiology and Health Information, Public Health Division of Gipuzkoa, Basque Regional Health Department, San Sebastian, Spain; 40 Clinical Gerontology Unit, School of Clinical Medicine, Addenbrooke’s Hospital, University of Cambridge, Cambridge, UK

**Keywords:** Diet, Nutrition, Epidemiology, Cohort study, Prostate cancer

## Abstract

**Purpose:**

The evidence from the literature regarding the association of dietary factors and risk of prostate cancer is inconclusive.

**Methods:**

A nutrient-wide association study was conducted to systematically and comprehensively evaluate the associations between 92 foods or nutrients and risk of prostate cancer in the European Prospective Investigation into Cancer and Nutrition (EPIC). Cox proportional hazard regression models adjusted for total energy intake, smoking status, body mass index, physical activity, diabetes and education were used to estimate hazard ratios and 95% confidence intervals for standardized dietary intakes. As in genome-wide association studies, correction for multiple comparisons was applied using the false discovery rate (FDR < 5%) method and suggested results were replicated in an independent cohort, the Netherlands Cohort Study (NLCS).

**Results:**

A total of 5916 and 3842 incident cases of prostate cancer were diagnosed during a mean follow-up of 14 and 20 years in EPIC and NLCS, respectively. None of the dietary factors was associated with the risk of total prostate cancer in EPIC (minimum FDR-corrected *P*, 0.37). Null associations were also observed by disease stage, grade and fatality, except for positive associations observed for intake of dry cakes/biscuits with low-grade and butter with aggressive prostate cancer, respectively, out of which the intake of dry cakes/biscuits was replicated in the NLCS.

**Conclusions:**

Our findings provide little support for an association for the majority of the 92 examined dietary factors and risk of prostate cancer. The association of dry cakes/biscuits with low-grade prostate cancer warrants further replication given the scarcity in the literature.

**Electronic supplementary material:**

The online version of this article (10.1007/s00394-019-02132-z) contains supplementary material, which is available to authorized users.

## Introduction

Prostate cancer is the most commonly diagnosed cancer in men residing in high-income countries [1]. Incidence rates of the disease, mostly at the localized stage, differ remarkably worldwide in large part because of the different uptake of prostate-specific antigen (PSA) test for screening but possibly also due to genetic and environmental factors [1, 2]. Among the environmental factors, nutrition-related factors such as obesity have been suggested to play a role in prostate cancer risk, but with different associations according to disease stage and grade [3–6]. However, the evidence for specific foods or nutrients affecting prostate cancer is inconsistent, and no specific dietary factors have been robustly associated with prostate cancer risk [3–5]. A potential reason for this inconsistency is that foods and nutrients are strongly correlated, and it is difficult to decipher their independent effects.

Therefore, we conducted a nutrient-wide association study (NWAS) to systematically and comprehensively evaluate the association between 92 foods or nutrients and risk of prostate cancer in the European Prospective Investigation into Cancer and Nutrition (EPIC) using proper adjustment for multiplicity of comparisons and replication of emerging findings in an independent population, the Netherlands Cohort Study (NLCS), as is commonly done in genome-wide association studies (GWAS). The NWAS method has been used to identify novel dietary risk associations for type 2 diabetes, blood pressure and cancer [7–10].

## Materials and methods

### Study population

EPIC is a large multi-center European prospective study aiming to identify the role of environmental, lifestyle and dietary factors on risk of cancer and other chronic diseases. Briefly, 521,324 participants, aged mostly between 35 and 70 years, were recruited from 1992 to 2000 from 23 centers across 10 European countries. More information about the EPIC cohort can be found elsewhere [11]. The current analysis included participants from eight countries, as EPIC centers in France and Norway recruited only women. Out of the 153,426 male participants in EPIC, 122,998 individuals were used in the current analysis after excluding men with prevalent cancer at recruitment except of non-melanoma skin cancer (*N* = 3972), those with lack of follow-up information (*N* = 1447) and lack of lifestyle and dietary information at recruitment (*N* = 2916), men with extreme values (top or bottom 1%) on the energy intake-to-energy requirement ratio (*N* = 2850), and men with missing values for the study confounders (*N* = 19,243). All the participants gave written informed consent, and the study was approved by the ethical review boards of the International Agency for Research on Cancer (IARC) and all local institutions.

NLCS is a prospective cohort study established in 1986 and includes 120,852 participants aged between 55 and 69 years recruited from 204 computerized population registries across the Netherlands, of which 58,279 were men [12]. The NLCS used a case–cohort approach for efficiency reasons, where a subcohort of 5000 participants, of which 2411 men, was selected at random immediately after baseline [12]. This subcohort has been followed up biannually to estimate the person-time at risk. Out of the 2411 male participants in the NLCS subcohort, 1961 individuals were used in the current analysis after excluding 75 men with prevalent cancer at recruitment, 279 men with incomplete or inconsistent dietary data, and 96 men with missing data on confounders. The NLCS was approved by the institutional review boards of the TNO Quality of Life research institute (Zeist, The Netherlands) and Maastricht University (Maastricht, The Netherlands).

### Assessment of dietary factors

The consumption of foods over the preceding 12 months was assessed at baseline in EPIC using country-specific food questionnaires including a range of 88–266 items. Questionnaires were self-administered, except in Greece, Spain and Ragusa in Italy, where interviewers were used. The EPIC Nutrient Database (ENDB) was used to calculate standardized nutrient intakes [13]. Further details on the diet questionnaires are described elsewhere [11]. In total, 92 dietary factors, of which 63 foods and 29 nutrients, that were available in at least six out of the eight countries were included in the current analysis.

In NLCS, food consumption in the previous 12 months was assessed at baseline using a semi-quantitative 150-item food frequency questionnaire, which has been validated and tested for reproducibility [14, 15]. The Dutch food composition table was used to convert the data from the questionnaire to nutrient intake [16].

### Assessment of prostate cancer

A total of 5916 men were diagnosed with first incident prostate cancer in EPIC based on the International Classification of Diseases 10th revision code (ICD-10: C61), the vast majority of which were adenocarcinomas (94%), a remaining 5% were unclassified tumors and 1% were other subtypes. Cancers were identified through population-based cancer registries in Denmark, Italy, The Netherlands, Spain, Sweden, and the United Kingdom (UK). Active follow-up, including direct or next of kin communication, or combination of different sources of ascertainment including health insurance records, regional health departments, municipality registries, hospital records and pathology registries were used in Greece and Germany. Of the 5916 incident prostate cancer cases, 3465 (58.5%) had stage information and 3742 (63.2%) had grade information. A total of 2321 tumors were classified as localized (i.e., tumor-node-metastasis [TNM] staging score of T0–T2 and N0/NX and M0, or stage coded in the recruitment center as localized) and 1144 as advanced prostate cancer (i.e., T3–T4 and/or N1–N3 and/or M1, or stage coded in the recruitment center as metastatic). Further, 3164 tumors were classified as low-grade (i.e., Gleason score of < 8, or grade coded as well differentiated or moderately differentiated) and 578 as high-grade prostate cancer (i.e., Gleason score ≥ 8, or grade coded as poorly differentiated or undifferentiated). Moreover, 1770 cancer cases were characterized as aggressive if they were recorded as advanced stage or high-grade cancer or had PSA value at diagnosis over 20 ng/mL. During the follow-up period, 709 fatal cases of prostate cancer were identified.

A total of 3842 cases of incident prostate cancer were diagnosed in NLCS through periodic record linkages to the Netherlands Cancer Registry. Information about the stage at diagnosis was available for 3563 (92.7%) cases, where 2312 cases were categorized as localized and 1251 as advanced. Information on prostate cancer differentiation grade was available for 3446 (89.6%) cases, of which 2593 were classified as low-grade and 853 as high-grade. Finally, 1667 cancer cases were characterized as aggressive.

### Statistical analysis

Each of the 92 dietary factors was individually explored in relation to prostate cancer risk in EPIC using Cox proportional hazard regression models. Age at entry was used as the time variable in all models. As age at exit was chosen either the age at cancer diagnosis or the age at death or age at the last follow-up, whichever happened first. The intake of nutrients was energy adjusted using the residual method in order to control for possible confounding due to total energy intake [17]. All dietary factors entered the models as standardized continuous variables to reflect associations per one standard deviation increase in consumption. As a sensitivity analysis, the dietary factors were also modeled as categorical variables using quartiles, but the results were very similar and therefore not reported herein.

The statistical models in EPIC were stratified by age at recruitment (< 40, 40–44.9, 45–49.9, 50–54.9, 55–59.9, 60–64.9, 65–69.9, 70–74.9, ≥ 75) and recruitment center in order to control for center specific differences like questionnaire design and follow-up procedures [18]. Covariates were selected a priori as potential confounders, which were total energy intake (kcal, continuous), smoking status (never, former, current), BMI (kg/m^2^, < 20, 20–22.9, 23–24.9, 25–29.9, 30–34.9, ≥ 35), physical activity (inactive, moderately inactive, moderately active, active) [19], diabetes history (no, yes), and education status (none/primary, technical/professional, secondary, longer). When we further adjusted for height, the results were similar and therefore height was not included in the final models. In the primary analysis participants with missing confounder values were excluded, but when the analysis was repeated using missing indicators identical results were observed (results not shown). In the NLCS, Prentice-weighted Cox proportional hazards regression models with robust estimation of standard errors were used to suit the case-cohort design [20]. The models in the NLCS were adjusted for the aforementioned confounders, and further for family history of prostate cancer (yes, no). The proportionality of the hazards was verified in both cohorts by checking the slope of the Schoenfeld residuals, and no violations were identified.

To account for multiple comparisons in the EPIC study, we estimated the false discovery rate (FDR) for each dietary factor. The FDR is the proportion of false positive results among the statistically significant results, and was computed using an analytical method described elsewhere [7, 8]. Briefly, a “null distribution” of *p* values was created by shuffling cancer status and years of follow-up and rerunning the Cox models. Then, the FDR was calculated as the ratio of the proportion of results that were nominally statistically significant at the 5% level in the null distribution and the proportion of nominally statistically significant results in the original analysis. As a sensitivity analysis, we calculated the FDR using the sequential *p* value approach proposed by Benjamini and Hochberg, but the obtained results were very similar [21]. Dietary factors with FDR value less than 5% were selected for replication in the NLCS study. Finally, a random-effects meta-analysis was performed to combine the results from the two cohorts and give an overall result. The analysis was performed using the R programming language and STATA (version 13).

## Results

After a mean follow-up time of 14 years for 122,998 men in EPIC, 5916 incident cases of total prostate cancer were identified. Table 1 shows the baseline characteristics of the study participants. In EPIC, the mean BMI was 26.6 kg/m^2^, about 30% were current smokers and had higher education levels while, over the half were physically active and only 4% had a history of diabetes. In NLCS, the mean BMI was 24.9 kg/m^2^, around 35% of the participants were smokers and 20% had received higher education. Moreover, the participants were spending about 81 min per day on average on non-occupational physical activities and about 3% of them had a history of diabetes.

**Table 1:** Distribution of demographic characteristics at baseline in EPIC and NLCS

Characteristics	EPIC	NLCS^a^
No. of participants	122,998	58,279
Mean age at recruitment (SD)	51.6 (9.8)	61.3 (4.2)
Mean BMI (SD)	26.6 (3.7)	24.9 (2.6)
Mean energy intake in kcals (SD)	2429 (663)	2164 (498)
% Current smokers	30.8	35.3
% Active/moderately active	50.9	
Mean physical activity, min/day (SD)		80.6 (67.5)
% History of diabetes	4.0	3.3
% Higher than secondary education	28.3	19.6

*EPIC* European Prospective Investigation into Cancer and Nutrition, *NLCS* Netherlands Cohort Study, *SD* standard deviation, *BMI* body mass index, *kcals* kilocalories

^a^Distribution data in NLCS are from the subcohort

**Fig. 1:**
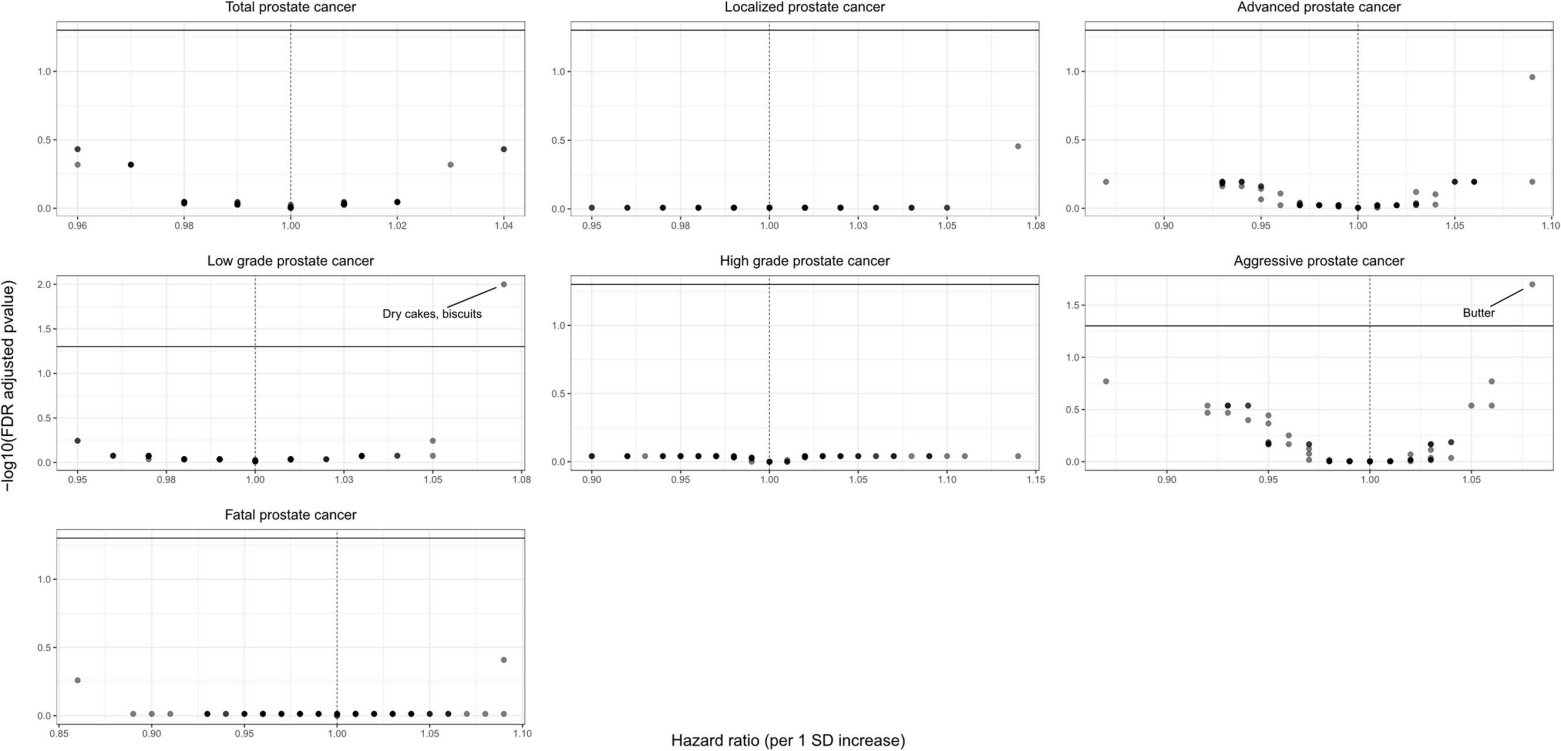
Volcano plot showing results from the nutrient-wide association study regarding the association between 92 dietary factors and prostate cancer risk overall and by stage, grade and fatality in the European Prospective Investigation into Cancer and Nutrition study. The *Y*-axis shows the false discovery rate (FDR) adjusted *P* values in −log10 scale from the Cox proportional hazards models for each dietary factor. The *X*-axis shows the estimated hazard ratio for each dietary factor per 1 standard deviation (SD) increase in consumption. The horizontal line represents the level of significance corresponding to FDR less than 5%. The models were adjusted for total energy intake (kcal, continuous); smoking status (never, former, current); BMI (< 20, 20–22.9, 23–24.9, 25–29.9, 30–34.9, ≥ 35 kg/m^2^); physical activity (inactive, moderately inactive, moderately active, active); diabetes history (no, yes); education status (none/primary, technical/professional, secondary, longer)

Of the 92 dietary factors that were evaluated in EPIC, three (dry cakes-biscuits, sauces, confectionary [non-chocolate]) were positively associated with total prostate cancer risk, while seven (total fruits, citrus fruits, vitamin B6, stone fruits, beer-cider, vitamin C, mushrooms) showed a protective association at the nominal statistical significance level (*P* ≤ 0.05) (Fig. 1 and supplemental Table 1). However, no dietary factor retained an association after correcting for multiple comparisons (smallest FDR *P*, 0.37). The results were similar and null associations were observed after correcting for multiple comparisons for almost all dietary variables and risk of prostate cancer by stage, grade or fatality with only two exceptions (Fig. 1 and supplemental Tables 2–7). Intakes of dry cakes/biscuits and butter were associated with a higher risk of low-grade (HR, 1.07; 95% CI 1.03–1.11; FDR *P*, 0.01) and aggressive prostate cancer (HR, 1.08; 95% CI, 1.04–1.13; FDR *P*, 0.02), respectively (Fig. 1 and supplemental Tables 4, 6). There was no evidence of heterogeneity of the latter two associations by prostate cancer grade, but evidence of heterogeneity was observed only for butter consumption by prostate cancer stage (*P*_hetereogeneity_, 0.02; HR, 0.98; 95% CI 0.94–1.02 for localized disease and HR, 1.09; 95% CI 1.03–1.15 for advanced disease). There was consistency in the results for both associations when they were analyzed separately in each EPIC country (Supplemental Fig. 1).

**Fig. 2:**
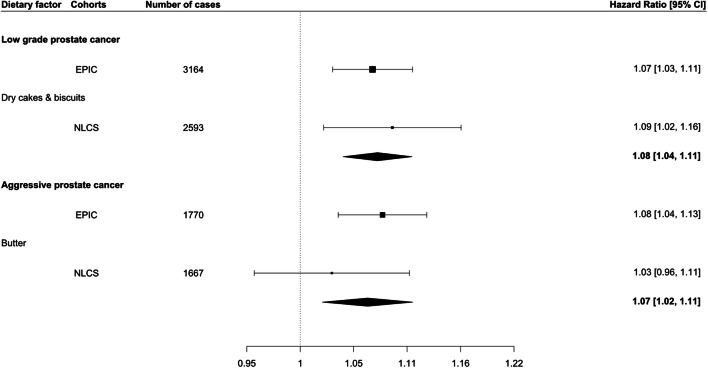
Forest plot showing the hazard ratios and 95% confidence intervals for the association of dry cakes/biscuits and butter consumption with the risk of low grade and aggressive prostate cancer, respectively, in the European Prospective Investigation into Cancer and Nutrition (EPIC) and the Netherlands Cohort Study (NLCS), as well as, the results from a random-effects meta-analysis between the two cohorts. The *X*-axis shows the estimated hazard ratio for each dietary factor for 1 standard deviation increase in consumption

In the NLCS, we then evaluated the two suggested associations from the analysis in EPIC (Fig. 2). The association for dry cakes/biscuits and risk of low-grade prostate cancer was replicated (HR 1.09; 95% CI 1.02–1.16), whereas the association for butter and aggressive prostate cancer risk was not (HR, 1.03; 95% CI 0.96–1.11). However, the association estimates in the NLCS were similar to the estimates observed in EPIC, and we conducted therefore a meta-analysis, where positive associations were observed for both foods (dry cakes/biscuits: HR, 1.08; 95% CI 1.04–1.11; butter: HR 1.07; 95% CI 1.02–1.11).

## Discussion

In the current study, we used the NWAS approach to evaluate the association between a large number of dietary factors and risk of total prostate cancer and cancer by stage, grade and fatality. After adjusting for multiple comparisons, no dietary variable was associated with risk of total prostate cancer or most other prostate cancer outcomes including mortality in EPIC. However, positive associations emerged for the consumption of dry cakes/biscuits and butter with risk of low-grade and aggressive prostate cancer, respectively, the first association of which was replicated in NLCS.

The current literature evidence supports the main findings of our analysis, as there is no consistent evidence for association between any dietary factor and risk of prostate cancer, which was confirmed by the World Cancer Research Fund (WCRF) Third Expert Report and a recent comprehensive review [3, 4]. The literature evidence for an association between dry cakes/biscuits and butter with risk of prostate cancer is very sparse. A report from the Malmö Diet and Cancer cohort showed that high intake of cakes and biscuits was associated with an increased risk of non-aggressive prostate cancer (top vs. bottom intake: HR 1.45; 95% CIs 1.03–2.02), and null associations were observed for aggressive or total prostate cancer risk [22]. This cohort included 8128 men, of whom 817 developed prostate cancer, and analyzed 16 dietary exposures related to carbohydrates, fiber and their food sources without correcting for multiple comparisons. In agreement, we observed in the current NWAS analysis that intake of dry cakes/biscuits was positively associated with low-grade disease after multiplicity correction and replication approaches, but also with localized and total prostate cancer risk before multiplicity correction. However, we observed null associations for intake of dry cakes/biscuits and risk of the more clinically important outcomes of advanced stage, high grade, aggressive and fatal prostate cancer. A potential explanation for the positive association between cakes and biscuits with low-grade prostate cancer could be the high concentration of refined carbohydrates in cakes and biscuits. High consumption of refined carbohydrates can lead to hyperinsulinemia followed by the activation of the insulin like growth factor (IGF)-1 axis and inflammatory pathways [23]. However, several prospective cohort studies have shown no association between glycaemic index or load and prostate cancer risk [24–26]. Another explanation could be the high levels of fat and especially saturated fat in cakes and biscuits, but null associations have been reported for total or saturated fat consumption in relation to risk of any prostate cancer outcome in the meta-analyses conducted by the WCRF Third Expert Report [3].

The current NWAS study reported a positive association between butter consumption and risk of aggressive prostate cancer after multiplicity correction and replication approaches, but also with advanced stage disease before multiplicity correction. The literature evidence is again very sparse, and a recent meta-analysis of two prospective studies found no association with total prostate cancer risk (high vs. low intake; RR 1.03; 95% CIs 0.89–1.20) in agreement with our findings [27]. A report from the Melbourne Collaborative Cohort Study observed no association between butter consumption and risk of aggressive prostate cancer (high vs. low intake; RR 1.03; 95% CIs 0.53–2.00), but included only 107 aggressive cases [28]. Another report from the Health Professionals Follow-up Study observed an increased risk of metastatic prostate cancer (249 cases; top vs. bottom consumption; RR 1.42; 95% CI 1.00–2.00), but this association was attenuated and lost statistical significance after additional controlling for saturated fat and a-linolenic acid [29]. It is likely that butter consumption reflects to an extent intake of dairy products, which have been associated with an increased risk of total prostate cancer in several prospective studies [27]. However, the association of dairy products with aggressive or fatal disease is inconsistent and received a weak evidence grading in a recent critical appraisal of the literature [4].

The strengths of the current study were the ability to systematically examine a large number of dietary factors in relation to all prostate cancer outcomes, including the most clinically relevant outcomes of aggressive or fatal disease, while taking into account the multiple comparisons by calculating the FDR and replicating results in an independent cohort, which provided further confidence in our findings. The NWAS approach necessitates the reporting of all results, and thus it addresses the issue of selective reporting of statistically significant results that is quite prevalent in observational epidemiology [8, 30]. Potential limitations of this study included the single dietary assessment at baseline and the use of self-reported questionnaires, which could lead to non-differential misclassification of dietary consumption and may drive the results towards the null. Men with any missing values for the study confounders (12.5%) were excluded from the analysis, which could lead to selection bias, but the excluded participants had a similar profile to those that remained, and the results were identical when the analysis was repeated using missing indicator values. Furthermore, it is possible that there might be an association for foods or nutrients that were not included in this analysis or for specific dietary patterns. Moreover, we did not account for the correlations between foods and nutrients as it would reduce the number of statistical comparisons and the corresponding statistical significance threshold. The number of fatal prostate cancer cases was relatively small despite the large number of total prostate cancer cases and the long follow-up period, and this analysis might lack statistical power. Finally, we cannot totally exclude the possibility of residual confounding, although we adjusted for several potential confounders.

In summary, no association was found for the majority of the 92 examined dietary factors and risk of prostate cancer. The associations of dry cakes/biscuits with low-grade prostate cancer and of butter with aggressive disease warrant further replication given the scarcity in the literature and the lack of clear mechanistic pathways.

## Electronic supplementary material

Below is the link to the electronic supplementary material.

Supplementary material 1 (PDF 180 kb)

Supplementary material 2 (PDF 520 kb)
