# Supplementary figures and images for: A nutrient-wide association study for risk of prostate cancer in the European Prospective Investigation into Cancer and Nutrition and the Netherlands Cohort Study
Source: Eur J Nutr. 2019 Nov 8;59(7):2929–37. doi: 10.1007/s00394-019-02132-z (PMC7501135; doi:10.1007/s00394-019-02132-z)

### Cakes & biscuits consumption

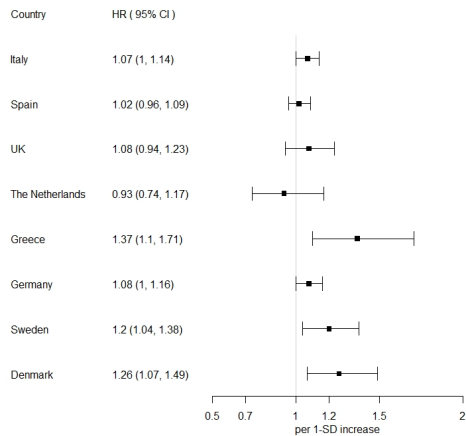

### Butter consumption

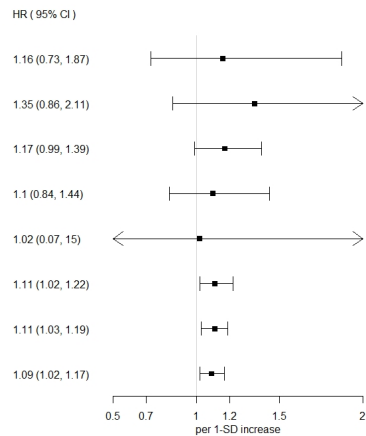

Supplement: Supplementary file 1 — Supplementary material 1 (PDF 180 kb) [file 394_2019_2132_MOESM1_ESM.pdf]
