# Supplementary material for: A nutrient-wide association study for risk of prostate cancer in the European Prospective Investigation into Cancer and Nutrition and the Netherlands Cohort Study
Source: Eur J Nutr. 2019 Nov 8;59(7):2929–37. doi: 10.1007/s00394-019-02132-z (PMC7501135; doi:10.1007/s00394-019-02132-z)
Supplement: Supplementary file 2 — Supplementary material 2 (PDF 520 kb) [file 394_2019_2132_MOESM2_ESM.pdf]

Supplemental table 1. Hazard ratios[a] and 95% CIs for the association of 92 food and nutrient intake in relation to total prostate cancer risk in EPIC				
**Dietary variables**	**HR**	**95% CI**	**P-value**	**FDR**
Dry cakes, biscuits[b]	1.04	1.01-1.08	0.01	0.37
Fruits	0.96	0.93-0.99	0.01	0.37
Citrus fruits	0.96	0.92-0.99	0.01	0.37
Sauces	1.04	1.01-1.07	0.02	0.37
Vitamin B6[a]	0.97	0.94-1.00	0.03	0.48
Stone fruits[b]	0.96	0.93-1.00	0.03	0.48
Confectionery (non-chocolate)[c]	1.03	1.00-1.06	0.04	0.48
Beer, cider	0.97	0.94-1.00	0.04	0.48
Vitamin C[a]	0.97	0.93-1.00	0.05	0.48
Mushrooms[b]	0.97	0.93-1.00	0.05	0.48
Banana	0.98	0.95-1.01	0.11	0.9
Crustaceans[c]	1.02	0.99-1.05	0.14	0.9
Mayonnaise[b]	1.02	0.99-1.05	0.16	0.90
Offal	0.98	0.95-1.01	0.18	0.90
Potassium[a]	0.98	0.95-1.01	0.19	0.90
Cakes, sweets (non-milk based)	1.02	0.99-1.05	0.19	0.90
Starch[a]	1.02	0.99-1.05	0.20	0.90
Cream puddings/ desserts[d]	0.98	0.95-1.01	0.21	0.90
Bread	1.02	0.99-1.06	0.22	0.90
Non-white bread[e]	1.02	0.99-1.06	0.22	0.9
Soft drinks	0.98	0.95-1.01	0.25	0.9
Calcium[a]	1.02	0.99-1.04	0.25	0.9
Butter	1.01	0.99-1.04	0.26	0.9
Salty biscuits, crackers	1.02	0.99-1.05	0.26	0.9
Stalk vegetables, sprouts[b]	0.98	0.95-1.01	0.27	0.9
Fish products[f]	1.01	0.99-1.04	0.28	0.9
Polyunsaturated fats	0.98	0.95-1.01	0.28	0.9
Soup[g]	0.98	0.95-1.01	0.28	0.9
Egg[b]	0.98	0.96-1.01	0.29	0.9
Fruit and vegetables juice	1.02	0.99-1.05	0.3	0.9
Fish	1.02	0.99-1.05	0.31	0.9
Fats (animal)	0.99	0.96-1.01	0.32	0.9
Margarine (vegetables)	0.99	0.96-1.01	0.32	0.9
Beef[h]	0.98	0.95-1.02	0.35	0.92
Magnesium[a]	0.98	0.95-1.02	0.35	0.92
Margarine	0.99	0.95-1.02	0.38	0.92
Ice cream	1.01	0.99-1.04	0.38	0.92
Fats (plant)	0.98	0.93-1.03	0.4	0.92
Pasta, rice, other grains	0.98	0.94-1.02	0.4	0.92
Cheese	1.01	0.98-1.05	0.41	0.92
Milk	1.01	0.98-1.04	0.42	0.92
Thiamin[a]	0.98	0.95-1.02	0.42	0.92
Vitamin D[a]	1.01	0.98-1.04	0.48	0.94
Poultry	0.99	0.96-1.02	0.5	0.94
Dietary fibre[a]	0.99	0.96-1.02	0.52	0.94
Root vegetables	0.99	0.97-1.02	0.54	0.94
Berries[i]	0.99	0.97-1.02	0.54	0.94
Vitamin E[a]	0.99	0.95-1.03	0.54	0.94
Total sugars[a]	0.99	0.96-1.02	0.55	0.94
Apple, pear	0.99	0.96-1.02	0.55	0.94

Spirits[j]	1.01	0.98-1.04	0.55	0.94
Onion, garlic[b]	0.99	0.95-1.03	0.57	0.94
Lean fish[k]	1.01	0.98-1.05	0.59	0.94
Protein (plant)[a]	0.99	0.96-1.03	0.59	0.94
Lamb[l]	1.01	0.98-1.04	0.59	0.94
Fatty fish[m]	1.01	0.98-1.03	0.6	0.94
Legumes[g]	1.01	0.97-1.06	0.61	0.94
Beta-carotene[a]	0.99	0.97-1.02	0.62	0.94
Saturated fats[a]	1.01	0.98-1.04	0.63	0.94
Riboflavin[a]	0.99	0.96-1.02	0.64	0.94
Retinol[a]	0.99	0.97-1.02	0.65	0.94
Coffee	0.99	0.96-1.03	0.66	0.94
Grain and pod vegetables[b]	0.99	0.95-1.03	0.67	0.94
Fortified wines[n]	1.01	0.98-1.03	0.67	0.94
Iron[a]	0.99	0.96-1.02	0.68	0.94
Carbohydrates[a]	1.01	0.98-1.04	0.69	0.94
Sugars (Sugar, honey, jam and syrup)	1.01	0.98-1.03	0.7	0.94
Monounsaturated fats[a]	1.01	0.97-1.05	0.7	0.94
Leafy vegetables[b]	0.99	0.95-1.03	0.72	0.94
Alcohol[a]	0.99	0.97-1.02	0.72	0.94
White bread[e]	1.01	0.97-1.05	0.72	0.94
Protein (animal)[a]	1.01	0.97-1.04	0.73	0.94
Tea[o]	1.00	0.98-1.03	0.74	0.94
Phosphorous[a]	1.00	0.97-1.04	0.78	0.97
Chocolate	1.00	0.97-1.03	0.79	0.97
Vitamin B12[a]	1.00	0.97-1.02	0.83	0.99
Liver[p]	1.00	0.98-1.03	0.83	0.99
Grapes[q]	1.00	0.96-1.03	0.84	0.99
Processed meat	1.00	0.96-1.03	0.85	0.99
Potatoes	1.00	0.97-1.03	0.86	0.99
Wine	1.00	0.97-1.03	0.89	0.99
Total proteins[a]	1.00	0.97-1.03	0.89	0.99
Breakfast cereals[f]	1.00	0.98-1.03	0.89	0.99
Cholesterol[a]	1.00	0.97-1.03	0.92	0.99
Yoghurt	1.00	0.97-1.02	0.94	0.99
Crispbread, rusks	1.00	0.97-1.04	0.94	0.99
Fruiting vegetables[b]	1.00	0.95-1.05	0.95	0.99
Nuts	1.00	0.97-1.04	0.96	0.99
Total fats[a]	1.00	0.97-1.03	0.97	0.99
Cabbage[b]	1.00	0.96-1.04	0.98	0.99
Red meat	1.00	0.97-1.03	0.99	0.99
Pork	1.00	0.97-1.03	0.99	0.99

[a] All dietary factors entered the models as standardized continuous variables and reflect associations per one standard deviation increase in consumption. Nutrient intakes were adjusted for total energy intake using the regression residual method. The models were adjusted for total energy intake (kcal, continuous); smoking status (never, former, current); BMI <20, 20-22.9, 23-24.9, 25-29.9, 30-34.9, ≥35kg/m^2); physical activity (inactive, moderately inactive, moderately active, active); diabetes history (no, yes); education status (none/primary, technical/professional, secondary, longer [including university]). They were further stratified by age at recruitment (<40, 40-44.9, 45-49.9, 50-54.9, 55-59.9, 60-64.9, 65-69.9, 70-74.9, ≥75) and recruitment center.

[b] Intake for leafy vegetables, fruiting vegetables, mushrooms, cabbage, grain and pod vegetables, onion and garlic, stalk vegetables, stone fruits, egg, mayonnaise and dry cakes/biscuits was missing for participants from Umeå (9.5% missing across EPIC)

[c] Intake of crustaceans and confectionary was missing for participants from Germany (17.2% missing across EPIC)

[d] Intake of cream puddings/desserts was missing for participants from Italy and Umeå (20.7% missing across EPIC)

[e] Intake of white and non-white bread was missing for participants from Greece (8.3% missing across EPIC)

[f] Intake of breakfast cereals and fish products was missing for participants from Italy (11.2% missing across EPIC)

[g] Intake of legumes and soup was missing for participants from Denmark (20.1% missing across EPIC)
[h] Intake of beef was missing for participants from Greece and Umeå (11.7% missing across EPIC)
[i] Intake of berries was missing for participants from UK (8% missing across EPIC)
[j] Intake of spirits was missing for participants from Ragusa (2.3% missing across EPIC)
[k] Intake of lean fish was missing for participants from Germany and Umeå (26.7% missing across EPIC)
[l] Intake of lamb was missing for participants from Italy (North), The Netherlands and Umeå (24.4% missing across EPIC)
[m] Intake of fatty fish was missing for participants from Potsdam (8.3% missing across EPIC)
[n] Intake of fortified wines was missing for participants from Ragusa and Umeå (11.7% missing across EPIC)
[o] Intake of tea was missing for participants from Malmö (7.4% missing across EPIC)
[p] Intake of liver was missing for participants from The Netherlands and Umeå (15.5% missing across EPIC)
[q] Intake of grapes was missing for participants from Umeå and Denmark (29.7% missing across EPIC)

Supplemental table 2. Hazard ratios[a] and 95% CIs from analyses of 92 foods and nutrient intake in relation to localised (stage) prostate cancer risk in EPIC				
**Dietary variables**	**HR**	**95% CI**	**P-value**	**FDR**
Dry cakes, biscuits[c]	1.07	1.02-1.11	0.004	0.35
Beer, cider	0.95	0.90-1.00	0.04	0.98
Crustaceans[b]	1.05	1.00-1.10	0.06	0.98
Fruit and vegetables juice	1.04	1.00-1.08	0.08	0.98
Citrus fruits	0.95	0.90-1.01	0.09	0.98
Fats (animal)	0.96	0.92-1.01	0.10	0.98
Thiamin[c]	1.05	0.98-1.12	0.14	0.98
Starch[a]	1.04	0.99-1.09	0.16	0.98
Mushrooms[c]	0.96	0.89-1.02	0.19	0.98
Berries[d]	1.02	0.99-1.06	0.22	0.98
Beef[e]	0.96	0.90-1.03	0.23	0.98
Bread	1.04	0.98-1.10	0.23	0.98
Fruit	0.97	0.92-1.02	0.26	0.98
Processed meat	1.03	0.98-1.09	0.28	0.98
Soft drinks	0.97	0.93-1.02	0.28	0.98
Non-white bread[f]	1.03	0.97-1.10	0.30	0.98
Saturated fats[c]	0.98	0.93-1.02	0.31	0.98
Fortified wines[g]	1.02	0.98-1.07	0.34	0.98
Stone fruits[c]	0.97	0.92-1.03	0.35	0.98
Banana	0.98	0.93-1.02	0.35	0.98
Pork	1.03	0.97-1.09	0.37	0.98
Riboflavin[a]	1.02	0.97-1.08	0.38	0.98
Butter	0.98	0.94-1.02	0.38	0.98
Nuts	0.97	0.91-1.04	0.38	0.98
cream puddings/desserts[h]	0.98	0.92-1.03	0.39	0.98
Total proteins[a]	1.02	0.97-1.07	0.40	0.98
Protein (animal)[a]	1.02	0.97-1.08	0.41	0.98
Spirits[i]	0.98	0.92-1.03	0.41	0.98
Monounsaturated fats[a]	1.02	0.96-1.09	0.46	0.98
Red meat	1.02	0.96-1.08	0.46	0.98
Carbohydrates[a]	1.02	0.97-1.07	0.51	0.98
Phosphorous[a]	1.02	0.97-1.07	0.52	0.98
Lean fish[j]	1.02	0.97-1.07	0.54	0.98
Cakes, sweets (non-milk based)	1.01	0.97-1.06	0.54	0.98
Fats (plant) [a]	1.02	0.95-1.10	0.55	0.98
Fish	1.01	0.97-1.06	0.55	0.98
Offal	0.98	0.94-1.04	0.55	0.98
White bread[f]	1.02	0.96-1.09	0.55	0.98
Iron[a]	1.01	0.97-1.06	0.55	0.98
Salty biscuits, crackers	1.02	0.96-1.07	0.56	0.98
Calcium[a]	1.01	0.97-1.06	0.57	0.98
Crispbread, rusks	0.99	0.94-1.04	0.57	0.98
Grapes[k]	0.98	0.92-1.05	0.58	0.98
Alcohol[a]	0.99	0.94-1.03	0.59	0.98
Pasta, rice, other grains	0.98	0.90-1.06	0.60	0.98
Milk	1.01	0.97-1.06	0.61	0.98
Tea[l]	1.01	0.96-1.06	0.62	0.98
Vitamin B12[a]	1.01	0.97-1.05	0.62	0.98
Margarine (vegetables)	1.01	0.96-1.06	0.63	0.98
Fruiting vegetable[c]	1.02	0.94-1.11	0.63	0.98

Onion, garlic[c]	0.98	0.93-1.05	0.63	0.98
Sauces	1.02	0.95-1.08	0.63	0.98
Vitamin D[a]	1.01	0.97-1.06	0.64	0.98
Lamb[m]	0.99	0.94-1.04	0.64	0.98
Grain and pod vegetables[c]	0.98	0.91-1.06	0.67	0.98
Chocolate	0.99	0.94-1.04	0.67	0.98
Coffee	0.99	0.93-1.05	0.68	0.98
Beta-carotene[a]	1.01	0.96-1.06	0.69	0.98
Poultry	0.99	0.95-1.04	0.71	0.98
Soup[n]	0.99	0.94-1.04	0.73	0.98
Breakfast cereals[o]	1.01	0.96-1.05	0.73	0.98
Total fats[a]	0.99	0.95-1.04	0.74	0.98
Egg[c]	0.99	0.95-1.04	0.75	0.98
Legumes[n]	1.01	0.95-1.07	0.76	0.98
Potatoes	1.01	0.96-1.05	0.77	0.98
Stalk vegetables, sprouts[c]	0.99	0.94-1.04	0.78	0.98
Polyunsaturated fats[c]	0.99	0.95-1.04	0.79	0.98
Margarine	1.01	0.95-1.07	0.80	0.98
Cholesterol[a]	1.01	0.95-1.06	0.81	0.98
Fish products[o]	0.99	0.95-1.04	0.82	0.98
Confectionery (non-chocolate)[b]	1.01	0.95-1.06	0.82	0.98
Cheese	0.99	0.94-1.05	0.83	0.98
Total sugars[a]	1.00	0.95-1.04	0.83	0.98
Dietary fibre[a]	1.00	0.96-1.05	0.84	0.98
Wine	1.00	0.96-1.05	0.86	0.98
Vitamin C[a]	1.00	0.94-1.05	0.86	0.98
Potassium[a]	1.00	0.96-1.05	0.87	0.98
Liver[p]	1.00	0.96-1.05	0.89	0.98
Sugars (Sugar, honey, jam and syrup)	1.00	0.96-1.04	0.90	0.98
Apple, pear	1.00	0.96-1.05	0.91	0.98
Vitamin B6[a]	1.00	0.95-1.05	0.91	0.98
Leafy vegetables[c]	1.00	0.95-1.06	0.92	0.98
Root vegetables	1.00	0.96-1.05	0.92	0.98
Vitamin E[a]	1.00	0.95-1.06	0.93	0.98
Protein (plant) [a]	1.00	0.94-1.06	0.94	0.98
Magnesium[a]	1.00	0.95-1.05	0.95	0.98
Ice cream	1.00	0.95-1.06	0.95	0.98
Yoghurt	1.00	0.96-1.04	0.96	0.98
Mayonnaise[c]	1.00	0.94-1.07	0.96	0.98
Retinol[a]	1.00	0.95-1.05	0.97	0.98
Fatty fish[q]	1.00	0.96-1.04	0.97	0.98
Cabbage[c]	1.00	0.93-1.07	0.98	0.98

[a] All dietary factors entered the models as standardized continuous variables and reflect associations per one standard deviation increase in consumption. Nutrient intakes were adjusted for total energy intake using the regression residual method. The models were adjusted for total energy intake (kcal, continuous); smoking status (never, former, current); BMI <20, 20-22.9, 23-24.9, 25-29.9, 30-34.9, ≥35kg/m^2); physical activity (inactive, moderately inactive, moderately active, active); diabetes history (no, yes); education status (none/primary, technical/professional, secondary, longer [including university]). They were further stratified by age at recruitment (<40, 40-44.9, 45-49.9, 50-54.9, 55-59.9, 60-64.9, 65-69.9, 70-74.9, ≥75) and recruitment center.
[b] Intake of crustaceans and confectionary was missing for participants from Germany (17.2% missing across EPIC)
[c] Intake for leafy vegetables, fruiting vegetables, mushrooms, cabbage, grain and pod vegetables, onion and garlic, stalk vegetables, stone fruits, egg, mayonnaise and dry cakes/biscuits was missing for participants from Umeå (9.5% missing across EPIC)
[d] Intake of berries was missing for participants from UK (8% missing across EPIC)
[e] Intake of beef was missing for participants from Greece and Umeå (11.7% missing across EPIC)
[f] Intake of white and non white bread was missing for participants from Greece (8.3% missing across EPIC)

[g] Intake of fortified wines was missing for participants from Ragusa and Umeå (11.7% missing across EPIC)
[h] Intake of cream puddings/desserts was missing for participants from Italy and Umeå (20.7% missing across EPIC)
[i] Intake of spirits was missing for participants from Ragusa (2.3% missing across EPIC)
[j] Intake of lean fish was missing for participants from Germany and Umeå (26.7% missing across EPIC)
[k] Intake of grapes was missing for participants from Umeå and Denmark (29.7% missing across EPIC)
[l] Intake of tea was missing for participants from Malmö (7.4% missing across EPIC)
[m] Intake of lamb was missing for participants from Italy (North), The Netherlands and Umeå (24.4% missing across EPIC)
[n] Intake of legumes and soup was missing for participants from Denmark (20.1% missing across EPIC)
[o] Intake of breakfast cereals and fish products was missing for participants from Italy (North) (11.2% missing across EPIC)
[p] Intake of liver was missing for participants from The Netherlands and Umeå (15.5% missing across EPIC)
[q] Intake of fatty fish was missing for participants from Potsdam (8.3% missing across EPIC)

Supplemental table 3. Hazard ratios[a] and 95% CIs from analyses of 92 foods and nutrient intake in relation to advanced (stage) prostate cancer risk in EPIC				
**Dietary variables**	**HR**	**95% CI**	**P-value**	**FDR**
Butter	1.09	1.03-1.15	0.001	0.11
Fats (plant)[b]	0.87	0.77-0.99	0.03	0.64
Alcohol[b]	1.06	1.00-1.12	0.05	0.64
Vitamin B6 [b]	0.93	0.86-1.00	0.05	0.64
Banana	0.93	0.87-1.00	0.06	0.64
Crispbread, rusks	1.09	1.00-1.19	0.06	0.64
Fatty fish[b]	1.06	1.00-1.12	0.07	0.64
Spirits[c]	1.05	1.00-1.10	0.07	0.64
Cream puddings/ desserts[d]	0.93	0.86-1.01	0.08	0.64
Total sugars	0.94	0.89-1.01	0.09	0.64
Polyunsaturated fats [b]	0.94	0.87-1.01	0.09	0.64
Poultry	0.94	0.88-1.01	0.09	0.64
Salty biscuits, crackers	1.05	0.99-1.12	0.1	0.64
Vitamin C [b]	0.93	0.86-1.02	0.11	0.64
Wine	1.05	0.99-1.13	0.11	0.64
Onion, garlic[e]	1.06	0.99-1.15	0.11	0.64
Coffee	1.06	0.99-1.13	0.12	0.64
Vitamin E [b]	0.93	0.85-1.02	0.13	0.65
Stone fruits[e]	0.93	0.85-1.02	0.14	0.66
Thiamin [b]	0.93	0.85-1.02	0.14	0.66
Fruit	0.94	0.87-1.02	0.16	0.69
Riboflavin [b]	0.95	0.88-1.02	0.18	0.69
Carbohydrates [b]	0.95	0.89-1.02	0.18	0.69
Pasta, rice, other grains	0.93	0.85-1.03	0.18	0.69
Offal	0.95	0.88-1.03	0.2	0.72
Fish products[f]	1.03	0.98-1.09	0.22	0.76
Tea[g]	0.96	0.91-1.02	0.23	0.78
Vitamin D [b]	1.04	0.97-1.11	0.24	0.79
Citrus fruits	0.95	0.87-1.04	0.27	0.86
Soft drinks	0.97	0.91-1.03	0.3	0.91
Total proteins [b]	0.97	0.90-1.03	0.32	0.92
Mayonnaise[e]	1.03	0.97-1.10	0.33	0.92
Saturated fats [b]	1.03	0.97-1.11	0.33	0.92
Potassium	0.97	0.90-1.04	0.36	0.94
Cheese	1.04	0.96-1.12	0.36	0.94
Stalk vegetables, sprouts[e]	1.03	0.97-1.10	0.37	0.94
Liver[h]	0.97	0.91-1.04	0.39	0.95
Margarine (vegetables)	0.97	0.90-1.04	0.4	0.95
Protein (animal) [b]	0.97	0.90-1.04	0.42	0.95
Fruit and vegetables juice	0.97	0.90-1.05	0.43	0.95
Mushrooms[e]	0.97	0.90-1.05	0.48	0.95
Grapes[i]	0.96	0.86-1.08	0.5	0.95
Phosphorous [b]	0.98	0.91-1.05	0.51	0.95
Lean fish[j]	0.97	0.88-1.07	0.52	0.95
Fish	1.02	0.95-1.11	0.53	0.95
Egg[e]	0.98	0.92-1.04	0.53	0.95
Root vegetables	0.98	0.92-1.04	0.53	0.95
Pork	0.98	0.91-1.05	0.54	0.95
Bread	1.03	0.95-1.11	0.54	0.95
Crustaceans[k]	0.97	0.89-1.06	0.54	0.95

Berries[l]	0.98	0.92-1.05	0.55	0.95
Vitamin B12 [b]	0.98	0.92-1.05	0.55	0.95
Yoghurt	0.98	0.92-1.04	0.56	0.95
Chocolate	0.98	0.92-1.05	0.56	0.95
Fats (animal) [b]	1.02	0.95-1.08	0.61	0.95
Retinol [b]	1.02	0.96-1.08	0.61	0.95
Non-white bread[m]	1.02	0.94-1.10	0.62	0.95
Beta-carotene [b]	0.99	0.93-1.04	0.64	0.95
Magnesium	0.98	0.91-1.06	0.64	0.95
Grain and pod vegetables [e]	0.98	0.89-1.08	0.66	0.95
Sugars (Sugar, honey, jam and syrup)	0.99	0.93-1.05	0.67	0.95
Milk	0.99	0.93-1.05	0.68	0.95
Monounsaturated fats [b]	0.98	0.89-1.08	0.68	0.95
Processed meat	0.98	0.91-1.07	0.71	0.95
Cholesterol [b]	0.99	0.92-1.06	0.71	0.95
Iron [b]	0.99	0.91-1.06	0.72	0.95
White bread[m]	0.98	0.89-1.09	0.72	0.95
Nuts	0.99	0.90-1.07	0.74	0.95
Dietary fibre [b]	0.99	0.93-1.05	0.74	0.95
Confectionery (non-chocolate)[k]	1.01	0.95-1.07	0.75	0.95
Ice cream	1.01	0.94-1.08	0.75	0.95
Apple, pear	1.01	0.95-1.07	0.76	0.95
Dry cakes, biscuits[e]	1.01	0.94-1.09	0.76	0.95
Leafy vegetables[e]	0.98	0.87-1.11	0.76	0.95
Calcium [b]	0.99	0.94-1.05	0.78	0.95
Legumes[n]	1.02	0.90-1.16	0.79	0.95
Beer, cider	1.01	0.95-1.07	0.80	0.95
Protein (plant) [b]	0.99	0.91-1.08	0.83	0.97
Margarine	0.99	0.91-1.08	0.84	0.97
Fortified wines[o]	1.01	0.95-1.06	0.84	0.97
Total fats [b]	0.99	0.93-1.06	0.86	0.97
Cabbage[e]	1.01	0.92-1.10	0.88	0.99
Breakfast cereals[f]	1.00	0.94-1.08	0.91	0.99
Starch [b]	1.00	0.93-1.08	0.93	0.99
Sauces	1.00	0.92-1.09	0.93	0.99
Red meat	1.00	0.93-1.08	0.95	0.99
Cakes, sweets (non-milk based)	1.00	0.93-1.08	0.95	0.99
Fruiting vegetable[e]	1.00	0.88-1.15	0.96	0.99
Soup[n]	1.00	0.92-1.08	0.96	0.99
Potatoes	1.00	0.94-1.07	0.97	0.99
Lamb[p]	1.00	0.92-1.08	0.98	0.99
Beef[q]	1.00	0.92-1.08	0.99	0.99
[a] All dietary factors entered the models as standardized continuous variables and reflect associations per one standard deviation increase in consumption. Nutrient intakes were adjusted for total energy intake using the regression residual method. The models were adjusted for total energy intake (kcal, continuous); smoking status (never, former, current); BMI <20, 20-22.9, 23-24.9, 25-29.9, 30-34.9, ≥35kg/m^2); physical activity (inactive, moderately inactive, moderately active, active); diabetes history (no, yes); education status (none/primary, technical/professional, secondary, longer [including university]). They were further stratified by age at recruitment (<40, 40-44.9, 45-49.9, 50-54.9, 55-59.9, 60-64.9, 65-69.9, 70-74.9, ≥75) and recruitment center.<br>[b] Intake of fatty fish was missing for participants from Potsdam (8.3% missing across EPIC)<br>[c] Intake of spirits was missing for participants from Ragusa (2.3% missing across EPIC)<br>[d] Intake of cream puddings/desserts was missing for participants from Italy and Umeå (20.7% missing across EPIC)<br>[e] Intake for leafy vegetables, fruiting vegetables, mushrooms, cabbage, grain and pod vegetables, onion and garlic, stalk vegetables, stone fruits, egg, mayonnaise and dry cakes/biscuits was missing for participants from Umeå (9.5% missing across EPIC)<br>[f] Intake of breakfast cereals and fish products was missing for participants from Italy (North) (11.2% missing across EPIC)				

[g] Intake of tea was missing for participants from Malmö (7.4% missing across EPIC)
[h] Intake of liver was missing for participants from The Netherlands and Umeå (15.5% missing across EPIC)
[i] Intake of grapes was missing for participants from Umeå and Denmark (29.7% missing across EPIC)
[j] Intake of lean fish was missing for participants from Germany and Umeå (26.7% missing across EPIC)
[k] Intake of crustaceans and confectionary was missing for participants from Germany (17.2% missing across EPIC)
[l] Intake of berries was missing for participants from UK (8% missing across EPIC)
[m] Intake of white and non-white bread was missing for participants from Greece (8.3% missing across EPIC)
[n] Intake of legumes and soup was missing for participants from Denmark (20.1% missing across EPIC)
[o] Intake of fortified wines was missing for participants from Ragusa and Umeå (11.7% missing across EPIC)
[p] Intake of lamb was missing for participants from Italy (North), The Netherlands and Umeå (24.4% missing across EPIC)
[q] Intake of beef was missing for participants from Greece and Umeå (11.7% missing across EPIC)

Supplemental table 4. Hazard ratios[a] and 95% CIs from analyses of 92 foods and nutrient intake in relation to low grade prostate cancer risk in EPIC				
**Dietary variables**	**HR**	**95% CI**	**P-value**	**FDR**
Dry cakes, biscuits[f]	1.07	1.03-1.11	0.0001	0.01
Offal	0.95	0.90-0.99	0.02	0.57
Crustaceans[b]	1.05	1.01-1.09	0.02	0.57
Beer, cider	0.95	0.91-0.99	0.02	0.57
Citrus fruits	0.96	0.91-1.00	0.05	0.84
White bread[c]	1.05	1.00-1.10	0.07	0.84
Fruit and vegetables juice	1.03	1.00-1.07	0.07	0.84
Fats (animal)[a]	0.97	0.93-1.00	0.08	0.84
Polyunsaturated fats[a]	0.97	0.93-1.01	0.1	0.84
Confectionery (non-chocolate)[b]	1.04	0.99-1.08	0.11	0.84
Bread	1.04	0.99-1.09	0.11	0.84
Liver[d]	0.97	0.93-1.01	0.12	0.84
Carbohydrates[a]	1.03	0.99-1.07	0.12	0.84
Starch[a]	1.03	0.99-1.08	0.14	0.84
Vitamin E[a]	0.96	0.92-1.01	0.15	0.84
Cream puddings/ desserts	0.97	0.92-1.01	0.15	0.84
Mushrooms[f]	0.96	0.91-1.02	0.16	0.84
Breakfast cereals[g]	1.03	0.99-1.06	0.17	0.85
Beef[h]	0.97	0.92-1.02	0.19	0.85
Stalk vegetables, sprouts[f]	0.97	0.93-1.02	0.2	0.85
Fish	1.03	0.99-1.07	0.2	0.85
Lamb[i]	1.03	0.98-1.07	0.21	0.85
Vitamin B6[a]	0.97	0.93-1.02	0.22	0.85
Root vegetables	0.97	0.93-1.02	0.23	0.85
Fruit	0.97	0.93-1.02	0.23	0.85
Egg[f]	0.98	0.94-1.02	0.27	0.92
Retinol[a]	0.98	0.94-1.02	0.3	0.92
Pasta, rice, other grains	0.97	0.92-1.03	0.33	0.92
Fatty fish[j]	1.02	0.98-1.05	0.35	0.92
Total fats[a]	0.98	0.94-1.02	0.35	0.92
Vitamin D[a]	1.02	0.98-1.06	0.36	0.92
Banana	0.98	0.94-1.02	0.37	0.92
Vitamin C[a]	0.98	0.94-1.03	0.37	0.92
Potassium[a]	0.98	0.94-1.02	0.37	0.92
Stone fruits[f]	0.98	0.94-1.02	0.38	0.92
Beta-carotene[a]	0.98	0.94-1.02	0.38	0.92
Magnesium[a]	0.98	0.94-1.03	0.43	0.92
Alcohol[a]	0.98	0.95-1.02	0.43	0.92
Berries[k]	1.01	0.98-1.05	0.44	0.92
Dietary fibre[a]	0.98	0.95-1.02	0.44	0.92
Leafy vegetables[f]	0.98	0.93-1.03	0.45	0.92
Soft drinks	0.98	0.94-1.03	0.48	0.92
Margarine (vegetables)	1.02	0.97-1.06	0.48	0.92
Fortified wines[l]	0.98	0.94-1.03	0.49	0.92
Grapes[m]	0.98	0.93-1.04	0.49	0.92
Tea[n]	1.01	0.97-1.06	0.52	0.92
Cabbage[f]	0.98	0.93-1.04	0.53	0.92
Onion, garlic[f]	0.98	0.93-1.04	0.54	0.92
Cakes, sweets (non-milk based)	1.01	0.97-1.05	0.55	0.92
Soup[o]	0.99	0.95-1.03	0.56	0.92

Grain and pod vegetables[f]	0.98	0.93-1.04	0.56	0.92
Saturated fats[a]	0.99	0.95-1.03	0.59	0.92
Iron[a]	0.99	0.95-1.03	0.59	0.92
Calcium[a]	1.01	0.97-1.05	0.6	0.92
Milk	1.01	0.97-1.05	0.62	0.92
Butter	1.01	0.97-1.05	0.62	0.92
Sugars (Sugar, honey, jam and syrup)	1.01	0.97-1.05	0.62	0.92
Cholesterol[a]	0.99	0.95-1.03	0.63	0.92
Riboflavin[a]	0.99	0.95-1.03	0.64	0.92
Mayonnaise[f]	1.01	0.96-1.06	0.67	0.92
Protein (animal)[a]	1.01	0.97-1.06	0.67	0.92
Lean fish[p]	1.01	0.97-1.06	0.68	0.92
Non-white bread[c]	1.01	0.96-1.06	0.71	0.92
Nuts	0.99	0.95-1.04	0.73	0.92
Total sugars[a]	1.01	0.97-1.05	0.73	0.92
Red meat	1.01	0.96-1.06	0.73	0.92
Ice cream	0.99	0.95-1.03	0.74	0.92
Fats (plant)[a]	0.99	0.93-1.06	0.74	0.92
Vitamin B12[a]	0.99	0.96-1.03	0.75	0.92
Total proteins[a]	1.01	0.97-1.05	0.75	0.92
Crispbread, rusks	0.99	0.95-1.04	0.76	0.92
Phosphorous[a]	0.99	0.95-1.04	0.76	0.92
Chocolate	1.01	0.97-1.05	0.76	0.92
Salty biscuits, crackers	1.01	0.96-1.05	0.77	0.92
Cheese	0.99	0.95-1.04	0.77	0.92
Processed meat	1.01	0.96-1.05	0.78	0.92
Sauces	1.01	0.96-1.06	0.78	0.92
Spirits[q]	0.99	0.96-1.04	0.78	0.92
Coffee	1.01	0.96-1.06	0.82	0.93
Pork	1.01	0.96-1.05	0.83	0.93
Apple, pear	1.00	0.97-1.04	0.83	0.93
Fruiting vegetable[f]	0.99	0.93-1.06	0.83	0.93
Protein (plant)[a]	1.00	0.95-1.04	0.85	0.95
Potatoes	1.00	0.96-1.05	0.87	0.96
Poultry	1.00	0.96-1.04	0.91	0.97
Yoghurt	1.00	0.97-1.04	0.92	0.97
Margarine	1.00	0.95-1.06	0.93	0.97
Thiamin[a]	1.00	0.95-1.05	0.95	0.97
Fish products[g]	1.00	0.96-1.04	0.95	0.97
Monounsaturated fats[a]	1.00	0.95-1.05	0.95	0.97
Legumes[o]	1.00	0.95-1.06	0.96	0.97
Wine	1.00	0.96-1.04	0.98	0.98

[a] All dietary factors entered the models as standardized continuous variables and reflect associations per one standard deviation increase in consumption. Nutrient intakes were adjusted for total energy intake using the regression residual method. The models were adjusted for total energy intake (kcal, continuous); smoking status (never, former, current); BMI <20, 20-22.9, 23-24.9, 25-29.9, 30-34.9, ≥35kg/m^2); physical activity (inactive, moderately inactive, moderately active, active); diabetes history (no, yes); education status (none/primary, technical/professional, secondary, longer [including university]). They were further stratified by age at recruitment (<40, 40-44.9, 45-49.9, 50-54.9, 55-59.9, 60-64.9, 65-69.9, 70-74.9, ≥75) and recruitment center.

[b] Intake of crustaceans and confectionary was missing for participants from Germany (17.2% missing across EPIC)

[c] Intake of white and non-white bread was missing for participants from Greece (8.3% missing across EPIC)

[d] Intake of liver was missing for participants from The Netherlands and Umeå (15.5% missing across EPIC)

[e] Intake of cream puddings/desserts was missing for participants from Italy and Umeå (20.7% missing across EPIC)

[f] Intake for leafy vegetables, fruiting vegetables, mushrooms, cabbage, grain and pod vegetables, onion and garlic, stalk vegetables, stone fruits, egg, mayonnaise and dry cakes/biscuits was missing for participants from Umeå (9.5% missing across EPIC)

[g] Intake of breakfast cereals and fish products was missing for participants from Italy (North) (11.2% missing across EPIC)
[h] Intake of beef was missing for participants from Greece and Umeå (11.7% missing across EPIC)
[i] Intake of lamb was missing for participants from Italy (North), The Netherlands and Umeå (24.4% missing across EPIC)
[j] Intake of fatty fish was missing for participants from Potsdam (8.3% missing across EPIC)
[k] Intake of berries was missing for participants from UK (8% missing across EPIC)
[l] Intake of fortified wines was missing for participants from Ragusa and Umeå (11.7% missing across EPIC)
[m] Intake of grapes was missing for participants from Umeå and Denmark (29.7% missing across EPIC)
[n] Intake of tea was missing for participants from Malmö (7.4% missing across EPIC)
[o] Intake of legumes and soup was missing for participants from Denmark (20.1% missing across EPIC)
[p] Intake of lean fish was missing for participants from Germany and Umeå (26.7% missing across EPIC)
[q] Intake of spirits was missing for participants from Ragusa (2.3% missing across EPIC)

Supplemental table 5. Hazard ratios[a] and 95% CIs from analyses of 92 foods and nutrient intake in relation to high grade prostate cancer risk in EPIC				
**Dietary variables**	**HR**	**95% CI**	**P-value**	**FDR**
Sugars (Sugar, honey, jam and syrup)	1.10	1.02-1.19	0.02	0.91
Non-white bread[b]	1.14	1.02-1.27	0.03	0.91
Leafy vegetables[c]	1.11	1.01-1.23	0.04	0.91
Vitamin B6[a]	0.90	0.82-1.00	0.04	0.91
Butter	1.07	0.99-1.16	0.07	0.91
Grapes[d]	1.09	0.99-1.19	0.07	0.91
Potassium [a]	0.92	0.83-1.01	0.09	0.91
Cakes, sweets (non-milk based)	1.08	0.99-1.18	0.1	0.91
Spirits[e]	1.07	0.99-1.15	0.1	0.91
Crustaceans[f]	0.90	0.80-1.02	0.1	0.91
Bread	1.09	0.98-1.22	0.12	0.91
Total proteins [a]	0.93	0.84-1.02	0.13	0.91
Pasta, rice, other grains	0.90	0.78-1.04	0.14	0.91
Stone fruits[c]	0.92	0.81-1.04	0.16	0.91
Sauces	1.07	0.96-1.20	0.21	0.91
Thiamin [a]	0.92	0.81-1.05	0.23	0.91
Protein (animal) [a]	0.94	0.85-1.04	0.24	0.91
Saturated fats [a]	1.06	0.96-1.16	0.26	0.91
Margarine (vegetables)	0.94	0.84-1.05	0.26	0.91
Confectionery (non-chocolate)[f]	0.95	0.86-1.04	0.26	0.91
Mayonnaise[c]	1.05	0.96-1.14	0.28	0.91
Fats (plant) [a]	0.92	0.78-1.07	0.29	0.91
Vitamin C [a]	0.94	0.84-1.05	0.29	0.91
Total fats [a]	1.05	0.96-1.15	0.3	0.91
Root vegetables	1.04	0.97-1.12	0.3	0.91
Poultry	0.95	0.87-1.05	0.31	0.91
Total sugars [a]	0.96	0.87-1.05	0.33	0.91
Fruiting vegetable[c]	1.09	0.92-1.29	0.33	0.91
Vitamin E [a]	1.06	0.94-1.19	0.33	0.91
Iron [a]	0.95	0.86-1.05	0.33	0.91
Potatoes	0.95	0.87-1.05	0.33	0.91
Lamb[g]	0.95	0.85-1.06	0.34	0.91
Red meat	0.95	0.85-1.06	0.34	0.91
Retinol [a]	1.04	0.96-1.13	0.34	0.91
Cheese	1.05	0.94-1.17	0.36	0.91
Beta-carotene [a]	1.03	0.96-1.11	0.37	0.91
Magnesium [a]	0.96	0.86-1.06	0.39	0.91
Legumes[h]	0.94	0.81-1.09	0.39	0.91
Pork	0.95	0.85-1.07	0.4	0.91
Dietary fibre [a]	1.04	0.95-1.13	0.4	0.91
Onion, garlic[c]	1.04	0.94-1.16	0.42	0.91
Fish	0.96	0.87-1.06	0.44	0.91
Riboflavin [a]	0.96	0.87-1.06	0.45	0.91
Milk	0.97	0.89-1.06	0.48	0.91
Apple, pear	1.03	0.95-1.11	0.48	0.91
Citrus fruits	0.96	0.86-1.07	0.49	0.91
Lean fish[i]	0.96	0.86-1.07	0.5	0.91
Carbohydrates [a]	0.97	0.88-1.06	0.51	0.91
Vitamin B12 [a]	0.97	0.89-1.06	0.52	0.91
Polyunsaturated fats [d]	1.03	0.94-1.14	0.52	0.91

Fats (animal) [a]	1.03	0.94-1.13	0.53	0.91
Nuts	1.03	0.93-1.14	0.54	0.91
Vitamin D [a]	0.97	0.87-1.07	0.54	0.91
Chocolate	0.97	0.87-1.08	0.55	0.91
Berries[j]	1.02	0.94-1.11	0.56	0.91
Soft drinks	0.97	0.89-1.07	0.57	0.91
Fruit	0.97	0.88-1.08	0.57	0.91
Alcohol [a]	1.02	0.94-1.11	0.58	0.91
Phosphorous [a]	0.97	0.88-1.08	0.6	0.91
Dry cakes, biscuits[c]	0.97	0.87-1.09	0.65	0.91
White bread[b]	0.97	0.85-1.10	0.65	0.91
Liver[k]	1.02	0.94-1.11	0.65	0.91
Fish products[l]	1.02	0.94-1.11	0.65	0.91
Monounsaturated fats [a]	1.03	0.90-1.17	0.65	0.91
Fortified wines[m]	1.02	0.94-1.10	0.66	0.91
Egg[c]	0.98	0.89-1.07	0.66	0.91
Banana	0.98	0.90-1.07	0.68	0.91
Coffee	1.02	0.92-1.13	0.68	0.91
Cream puddings/ desserts[n]	0.98	0.89-1.08	0.68	0.91
Wine	0.98	0.90-1.08	0.73	0.93
Mushrooms[c]	0.98	0.89-1.09	0.73	0.93
Tea[o]	0.99	0.91-1.07	0.74	0.93
Margarine	0.98	0.87-1.11	0.74	0.93
Starch [a]	1.02	0.92-1.12	0.75	0.93
Salty biscuits, crackers	0.98	0.88-1.10	0.76	0.93
Beer, cider	0.99	0.90-1.08	0.77	0.93
Grain and pod vegetables[c]	1.02	0.91-1.14	0.78	0.93
Protein (plant) [a]	0.99	0.88-1.10	0.8	0.95
Calcium [a]	0.99	0.91-1.08	0.82	0.95
Offal	1.01	0.92-1.11	0.84	0.97
Cholesterol [a]	1.01	0.92-1.11	0.88	1
Cabbage[c]	1.01	0.90-1.12	0.9	1
Stalk vegetables, sprouts[c]	1.01	0.92-1.11	0.91	1
Ice cream	0.99	0.90-1.10	0.91	1
Soup[h]	1.00	0.91-1.11	0.93	1
Breakfast cereals[l]	1.00	0.91-1.09	0.94	1
Crispbread, rusks	1.00	0.88-1.13	0.95	1
Processed meat	1.00	0.88-1.12	0.96	1
Fatty fish[p]	1.00	0.92-1.09	0.97	1
Beef[q]	1.00	0.90-1.12	0.98	1
Yoghurt	1.00	0.92-1.08	0.99	1
Fruit and vegetables juice	1.00	0.89-1.12	1	1

[a] All dietary factors entered the models as standardized continuous variables and reflect associations per one standard deviation increase in consumption. Nutrient intakes were adjusted for total energy intake using the regression residual method. The models were adjusted for total energy intake (kcal, continuous); smoking status (never, former, current); BMI <20, 20-22.9, 23-24.9, 25-29.9, 30-34.9, ≥35kg/m^2); physical activity (inactive, moderately inactive, moderately active, active); diabetes history (no, yes); education status (none/primary, technical/professional, secondary, longer [including university]). They were further stratified by age at recruitment (<40, 40-44.9, 45-49.9, 50-54.9, 55-59.9, 60-64.9, 65-69.9, 70-74.9, ≥75) and recruitment center.

[b] Intake of white and non-white bread was missing for participants from Greece (8.3% missing across EPIC)

[c] Intake for leafy vegetables, fruiting vegetables, mushrooms, cabbage, grain and pod vegetables, onion and garlic, stalk vegetables, stone fruits, egg, mayonnaise and dry cakes/biscuits was missing for participants from Umeå (9.5% missing across EPIC)

[d] Intake of grapes was missing for participants from Umeå and Denmark (29.7% missing across EPIC)

[e] Intake of spirits was missing for participants from Ragusa (2.3% missing across EPIC)

[f] Intake of crustaceans and confectionary was missing for participants from Germany (17.2% missing across EPIC)

[g] Intake of lamb was missing for participants from Italy (North), The Netherlands and Umeå (24.4% missing across EPIC)
[h] Intake of legumes and soup was missing for participants from Denmark (20.1% missing across EPIC)
[i] Intake of lean fish was missing for participants from Germany and Umeå (26.7% missing across EPIC)
[j] Intake of berries was missing for participants from UK (8% missing across EPIC)
[k] Intake of liver was missing for participants from The Netherlands and Umeå (15.5% missing across EPIC)
[l] Intake of breakfast cereals and fish products was missing for participants from Italy (North) (11.2% missing across EPIC)
[m] Intake of fortified wines was missing for participants from Ragusa and Umeå (11.7% missing across EPIC)
[n] Intake of cream puddings/desserts was missing for participants from Italy and Umeå (20.7% missing across EPIC)
[o] Intake of tea was missing for participants from Malmö (7.4% missing across EPIC)
[p] Intake of fatty fish was missing for participants from Potsdam (8.3% missing across EPIC)
[q] Intake of beef was missing for participants from Greece and Umeå (11.7% missing across EPIC)

Supplemental table 6. Hazard ratios[a] and 95% CIs from analyses of 92 foods and nutrient intake in relation to aggressive[b] prostate cancer risk in EPIC				
**Dietary variables**	**HR**	**95% CI**	**P-value**	**FDR**
Butter	1.08	1.04-1.13	0.0002	0.02
Spirits[c]	1.06	1.02-1.10	0.01	0.17
Fats (plant)[a]	0.87	0.80-0.96	0.01	0.17
Vitamin C [a]	0.92	0.86-0.98	0.01	0.29
Polyunsaturated fats [a]	0.93	0.88-0.99	0.02	0.29
Fruit	0.93	0.87-0.99	0.02	0.29
Salty biscuits, crackers	1.06	1.01-1.11	0.03	0.29
Alcohol [a]	1.05	1.01-1.11	0.03	0.29
Vitamin B6 [a]	0.94	0.89-0.99	0.03	0.29
Citrus fruits	0.93	0.87-0.99	0.03	0.29
Margarine (vegetables)	0.94	0.88-1.00	0.03	0.29
Vitamin E [a]	0.93	0.86-1.00	0.05	0.34
Pasta, rice, other grains	0.92	0.85-1.00	0.05	0.34
Banana	0.95	0.90-1.00	0.05	0.36
Cream puddings/ desserts[d]	0.94	0.89-1.00	0.07	0.4
Soft drinks	0.95	0.90-1.00	0.07	0.43
Carbohydrates [a]	0.96	0.91-1.01	0.1	0.56
Retinol [a]	1.04	0.99-1.08	0.13	0.65
Saturated fats [a]	1.04	0.99-1.10	0.14	0.65
Margarine	0.95	0.89-1.02	0.14	0.65
Stone fruits[e]	0.95	0.89-1.02	0.15	0.66
Protein (plant) [a]	0.95	0.89-1.02	0.17	0.68
Poultry	0.96	0.91-1.02	0.18	0.68
Thiamin [a]	0.95	0.88-1.03	0.19	0.68
Fats (animal) [a]	1.03	0.98-1.09	0.2	0.68
Sugars (Sugar, honey, jam and syrup)	1.03	0.98-1.08	0.21	0.68
Grain and pod vegetables[e]	0.95	0.88-1.03	0.21	0.68
Potassium [a]	0.97	0.91-1.02	0.22	0.68
Dietary fibre [a]	0.97	0.92-1.02	0.22	0.68
Mayonnaise[e]	1.03	0.98-1.09	0.22	0.68
Total sugars [a]	0.97	0.92-1.02	0.25	0.75
Wine	1.03	0.98-1.09	0.27	0.77
Total proteins [a]	0.97	0.92-1.03	0.3	0.84
Fortified wines[f]	1.02	0.98-1.06	0.31	0.85
Coffee	1.03	0.97-1.09	0.35	0.92
Leafy vegetables[e]	1.04	0.96-1.12	0.36	0.92
Mushrooms[e]	0.97	0.92-1.03	0.39	0.96
Starch [a]	0.98	0.92-1.03	0.41	0.96
Onion, garlic[e]	1.03	0.96-1.09	0.41	0.96
Cheese	1.03	0.96-1.09	0.42	0.96
Nuts	1.02	0.96-1.09	0.44	0.96
Non-white bread[g]	1.03	0.96-1.10	0.44	0.96
Fish products[h]	1.02	0.97-1.06	0.45	0.96
Fatty fish[i]	1.02	0.97-1.07	0.46	0.96
Offal	0.98	0.93-1.04	0.48	0.98
Magnesium [a]	0.98	0.93-1.04	0.5	0.99
Cholesterol [a]	1.02	0.96-1.07	0.53	0.99
Root vegetables	0.99	0.94-1.03	0.54	0.99
Beta-carotene [a]	0.99	0.94-1.03	0.58	0.99
Fruit and vegetables juice	0.98	0.92-1.05	0.6	0.99

Beer, cider	1.01	0.97-1.06	0.63	0.99
Soup[j]	0.99	0.93-1.05	0.64	0.99
Iron [a]	0.99	0.93-1.05	0.64	0.99
Breakfast cereals[h]	1.01	0.96-1.06	0.64	0.99
Chocolate	0.99	0.93-1.04	0.65	0.99
White bread[g]	0.98	0.91-1.06	0.66	0.99
Legumes[j]	0.98	0.90-1.07	0.67	0.99
Bread	1.01	0.95-1.08	0.69	0.99
Calcium [a]	1.01	0.96-1.06	0.69	0.99
Stalk vegetables, sprouts[e]	1.01	0.96-1.07	0.7	0.99
Yoghurt	1.01	0.96-1.06	0.7	0.99
Confectionery (non-chocolate)[k]	1.01	0.96-1.06	0.7	0.99
Milk	1.01	0.96-1.06	0.7	0.99
Pork	0.99	0.93-1.05	0.71	0.99
Tea[l]	0.99	0.94-1.04	0.71	0.99
Lean fish[m]	0.99	0.92-1.06	0.72	0.99
Beef[n]	0.99	0.93-1.06	0.74	0.99
Cakes, sweets (non-milk based)	1.01	0.95-1.07	0.74	0.99
Potatoes	0.99	0.94-1.05	0.76	0.99
Protein (animal) [a]	0.99	0.94-1.05	0.8	0.99
Berries[o]	1.01	0.96-1.06	0.81	0.99
Monounsaturated fats [a]	0.99	0.92-1.07	0.81	0.99
Ice cream	0.99	0.94-1.05	0.81	0.99
Red meat	0.99	0.93-1.06	0.81	0.99
Processed meat	1.01	0.94-1.08	0.85	0.99
Grapes[p]	0.99	0.92-1.07	0.86	0.99
Lamb[q]	0.99	0.93-1.06	0.86	0.99
Egg[e]	1.00	0.95-1.05	0.86	0.99
Sauces	1.01	0.94-1.08	0.87	0.99
Fish	1.00	0.94-1.06	0.87	0.99
Apple, pear	1.00	0.95-1.05	0.88	0.99
Liver[r]	1.00	0.95-1.05	0.91	0.99
Crustaceans[k]	1.00	0.94-1.06	0.92	0.99
Cabbage[e]	1.00	0.93-1.07	0.94	0.99
Vitamin D [a]	1.00	0.95-1.06	0.95	0.99
Fruiting vegetable[e]	1.00	0.90-1.11	0.95	0.99
Dry cakes, biscuits[e]	1.00	0.94-1.06	0.97	0.99
Riboflavin [a]	1.00	0.94-1.06	0.97	0.99
Crispbread, rusks	1.00	0.93-1.07	0.98	0.99
Vitamin B12 [a]	1.00	0.95-1.05	0.98	0.99
Total fats [a]	1.00	0.95-1.05	0.98	0.99
Phosphorous [a]	1.00	0.94-1.06	1	1

[a] All dietary factors entered the models as standardized continuous variables and reflect associations per one standard deviation increase in consumption. Nutrient intakes were adjusted for total energy intake using the regression residual method. The models were adjusted for total energy intake (kcal, continuous); smoking status (never, former, current); BMI <20, 20-22.9, 23-24.9, 25-29.9, 30-34.9, ≥35kg/m^2); physical activity (inactive, moderately inactive, moderately active, active); diabetes history (no, yes); education status (none/primary, technical/professional, secondary, longer [including university]). They were further stratified by age at recruitment (<40, 40-44.9, 45-49.9, 50-54.9, 55-59.9, 60-64.9, 65-69.9, 70-74.9, ≥75) and recruitment center.

[b] Prostate cancers with either advanced stage or high grade or PSA>20ng/mL at recruitment

[c] Intake of spirits was missing for participants from Ragusa (2.3% missing across EPIC)

[d] Intake of cream puddings/desserts was missing for participants from Italy and Umeå (20.7% missing across EPIC)

[e] Intake for leafy vegetables, fruiting vegetables, mushrooms, cabbage, grain and pod vegetables, onion and garlic, stalk vegetables, stone fruits, egg, mayonnaise and dry cakes/biscuits was missing for participants from Umeå (9.5% missing across EPIC)

[f] Intake of fortified wines was missing for participants from Ragusa and Umeå (11.7% missing across EPIC)

[g] Intake of white and non-white bread was missing for participants from Greece (8.3% missing across EPIC)
[h] Intake of breakfast cereals and fish products was missing for participants from Italy (North) (11.2% missing across EPIC)
[i] Intake of fatty fish was missing for participants from Potsdam (8.3% missing across EPIC)
[j] Intake of legumes and soup was missing for participants from Denmark (20.1% missing across EPIC)
[k] Intake of crustaceans and confectionary was missing for participants from Germany (17.2% missing across EPIC)
[l] Intake of tea was missing for participants from Malmö (7.4% missing across EPIC)
[m] Intake of lean fish was missing for participants from Germany and Umeå (26.7% missing across EPIC)
[n] Intake of beef was missing for participants from Greece and Umeå (11.7% missing across EPIC)
[o] Intake of berries was missing for participants from UK (8% missing across EPIC)
[p] Intake of grapes was missing for participants from Umeå and Denmark (29.7% missing across EPIC)
[q] Intake of lamb was missing for participants from Italy (North), The Netherlands and Umeå (24.4% missing across EPIC)
[r] Intake of liver was missing for participants from The Netherlands and Umeå (15.5% missing across EPIC)

Supplemental table 7. Hazard ratios[a] and 95% CIs from analyses of 92 foods and nutrient intake in relation to fatal prostate cancer risk in EPIC				
**Dietary variables**	**HR**	**95% CI**	**P-value**	**FDR**
Spirits[b]	1.09	1.03-1.15	0.004	0.39
Chocolate	0.86	0.77-0.97	0.01	0.55
Citrus fruits	0.89	0.79-0.99	0.04	0.97
Fish products[c]	1.06	1.00-1.13	0.04	0.97
Margarine	0.91	0.83-1.00	0.06	0.97
Bread	1.09	0.98-1.20	0.1	0.97
Onion, garlic[d]	1.08	0.98-1.18	0.11	0.97
Stone fruits[d]	0.90	0.79-1.02	0.11	0.97
Fruit	0.93	0.84-1.02	0.13	0.97
Fortified wines[e]	0.93	0.86-1.02	0.13	0.97
Vitamin C[a]	0.93	0.84-1.03	0.14	0.97
Cream puddings/ desserts[f]	0.94	0.86-1.02	0.15	0.97
Alcohol[a]	1.06	0.98-1.14	0.17	0.97
Vitamin B6[a]	0.94	0.86-1.03	0.18	0.97
Non-white bread[g]	1.07	0.97-1.19	0.19	0.97
Beef[h]	0.93	0.83-1.04	0.2	0.97
Ice cream	0.95	0.87-1.03	0.22	0.97
Yoghurt	0.95	0.88-1.03	0.23	0.97
Starch[a]	1.06	0.96-1.16	0.23	0.97
Coffee	0.95	0.87-1.04	0.24	0.97
Grapes[i]	1.05	0.96-1.14	0.27	0.97
Total sugars[a]	0.95	0.88-1.04	0.28	0.97
Stalk vegetables, sprouts[d]	1.04	0.97-1.12	0.29	0.97
Sauces	1.05	0.96-1.14	0.3	0.97
Berries[j]	0.96	0.88-1.04	0.3	0.97
Breakfast cereals[c]	1.03	0.97-1.08	0.31	0.97
Milk	1.04	0.97-1.11	0.32	0.97
Total fats[a]	0.96	0.88-1.04	0.34	0.97
Polyunsaturated fats[a]	0.96	0.87-1.05	0.36	0.97
Tea[k]	0.96	0.89-1.05	0.39	0.97
Red meat	0.96	0.87-1.06	0.39	0.97
Egg[d]	1.03	0.96-1.11	0.39	0.97
Cakes, sweets (non-milk based)	1.04	0.95-1.14	0.39	0.97
Protein (plant)	1.04	0.94-1.15	0.4	0.97
Potassium[a]	0.96	0.88-1.05	0.4	0.97
Butter	1.03	0.96-1.11	0.41	0.97
Crustaceans[l]	0.96	0.87-1.06	0.41	0.97
Saturated fats[a]	0.97	0.89-1.05	0.43	0.97
Iron[a]	1.04	0.95-1.13	0.44	0.97
Retinol[a]	0.97	0.90-1.05	0.46	0.97
Wine	1.03	0.94-1.13	0.47	0.97
Salty biscuits, crackers	1.03	0.95-1.12	0.49	0.97
Fruiting vegetable[d]	1.05	0.91-1.22	0.5	0.97
Cholesterol[a]	1.03	0.95-1.12	0.5	0.97
Dry cakes, biscuits[d]	1.03	0.94-1.14	0.52	0.97
Offal	0.97	0.90-1.06	0.53	0.97
Soft drinks	0.97	0.90-1.06	0.53	0.97
Protein (animal) [a]	0.97	0.88-1.07	0.54	0.97
Mushrooms[d]	1.03	0.94-1.12	0.56	0.97
Fruit and vegetables juice	1.03	0.93-1.14	0.57	0.97

Poultry	0.98	0.90-1.06	0.6	0.97
Margarine (vegetables)	0.98	0.91-1.06	0.61	0.97
Calcium[a]	1.02	0.95-1.10	0.62	0.97
Monounsaturated fats[a]	0.97	0.86-1.09	0.64	0.97
Banana	0.98	0.91-1.06	0.64	0.97
Total proteins[a]	0.98	0.90-1.07	0.65	0.97
Nuts	0.98	0.88-1.08	0.66	0.97
Confectionery (non-chocolate)[l]	1.01	0.95-1.09	0.68	0.97
White bread[g]	1.03	0.90-1.17	0.69	0.97
Lamb[m]	0.98	0.89-1.08	0.7	0.97
Dietary fibre[a]	1.02	0.94-1.10	0.7	0.97
Pork	0.98	0.90-1.07	0.71	0.97
Phosphorous[a]	1.02	0.93-1.11	0.71	0.97
Magnesium[a]	0.99	0.90-1.08	0.78	0.97
Grain and pod vegetables[d]	0.99	0.89-1.09	0.79	0.97
Vitamin D[a]	1.01	0.93-1.10	0.79	0.97
Lean fish[n]	1.01	0.92-1.12	0.79	0.97
Thiamin[a]	1.02	0.91-1.13	0.79	0.97
Leafy vegetables[d]	1.02	0.90-1.15	0.81	0.97
Fats (plant) [a]	0.98	0.86-1.13	0.82	0.97
Legumes[o]	1.01	0.89-1.16	0.84	0.97
Vitamin E[a]	0.99	0.88-1.11	0.84	0.97
Cheese	1.01	0.91-1.11	0.85	0.97
Fats (animal) [a]	1.01	0.93-1.09	0.85	0.97
Sugars (Sugar, honey, jam and syrup)	1.01	0.93-1.09	0.85	0.97
Vitamin B12[a]	0.99	0.93-1.06	0.86	0.97
Riboflavin[a]	0.99	0.91-1.08	0.87	0.97
Apple, pear	1.01	0.93-1.09	0.87	0.97
Soup[o]	0.99	0.91-1.09	0.87	0.97
Beer, cider	1.01	0.93-1.09	0.89	0.97
Liver[p]	1.01	0.93-1.08	0.89	0.97
Crispbread, rusks	1.01	0.90-1.13	0.89	0.97
Pasta, rice, other grains	1.01	0.89-1.15	0.91	0.97
Potatoes	1.00	0.92-1.08	0.91	0.97
Fatty fish[q]	1.00	0.93-1.08	0.92	0.97
Processed meat	0.99	0.90-1.11	0.92	0.97
Carbohydrates[a]	1.00	0.91-1.09	0.93	0.97
Mayonnaise[d]	1.00	0.93-1.08	0.93	0.97
Cabbage[d]	1.00	0.91-1.09	0.94	0.97
Beta-carotene[a]	1.00	0.94-1.07	0.95	0.97
Root vegetables	1.00	0.93-1.07	0.99	1
Fish	1.00	0.91-1.09	1	1

[a] All dietary factors entered the models as standardized continuous variables and reflect associations per one standard deviation increase in consumption. Nutrient intakes were adjusted for total energy intake using the regression residual method. The models were adjusted for total energy intake (kcal, continuous); smoking status (never, former, current); BMI <20, 20-22.9, 23-24.9, 25-29.9, 30-34.9, ≥35kg/m^2); physical activity (inactive, moderately inactive, moderately active, active); diabetes history (no, yes); education status (none/primary, technical/professional, secondary, longer [including university]). They were further stratified by age at recruitment (<40, 40-44.9, 45-49.9, 50-54.9, 55-59.9, 60-64.9, 65-69.9, 70-74.9, ≥75) and recruitment center.

[b] Intake of spirits was missing for participants from Ragusa (2.3% missing across EPIC)

[c] Intake of breakfast cereals and fish products was missing for participants from Italy (North) (11.2% missing across EPIC)

[d] Intake for leafy vegetables, fruiting vegetables, mushrooms, cabbage, grain and pod vegetables, onion and garlic, stalk vegetables, stone fruits, egg, mayonnaise and dry cakes/biscuits was missing for participants from Umeå (9.5% missing across EPIC)

[e] Intake of fortified wines was missing for participants from Ragusa and Umeå (11.7% missing across EPIC)

[f] Intake of cream puddings/desserts was missing for participants from Italy and Umeå (20.7% missing across EPIC)

[g] Intake of white and non-white bread was missing for participants from Greece (8.3% missing across EPIC)
[h] Intake of beef was missing for participants from Greece and Umeå (11.7% missing across EPIC)
[i] Intake of grapes was missing for participants from Umeå and Denmark (29.7% missing across EPIC)
[j] Intake of berries was missing for participants from UK (8% missing across EPIC)
[k] Intake of tea was missing for participants from Malmö (7.4% missing across EPIC)
[l] Intake of crustaceans and confectionary was missing for participants from Germany (17.2% missing across EPIC)
[m] Intake of lamb was missing for participants from Italy (North), The Netherlands and Umeå (24.4% missing across EPIC)
[n] Intake of lean fish was missing for participants from Germany and Umeå (26.7% missing across EPIC)
[o] Intake of legumes and soup was missing for participants from Denmark (20.1% missing across EPIC)
[p] Intake of liver was missing for participants from The Netherlands and Umeå (15.5% missing across EPIC)
[q] Intake of fatty fish was missing for participants from Potsdam (8.3% missing across EPIC)
